# Exploring the prognostic significance of immunogenic cell death‐related genes as risk biomarkers in cervical cancer

**DOI:** 10.1002/iid3.1260

**Published:** 2024-06-11

**Authors:** Shuangmei Jin, Jingdong Chen

**Affiliations:** ^1^ Department of Gynaecology and Obstetrics, Bishan Hospital of Chongqing Bishan Hospital of Chongqing Medical University Chongqing China

**Keywords:** cervical cancer, immunogenic cell death, immunotherapy, prognosis, tumor immune infiltration

## Abstract

**Background:**

Immunogenic cell death (ICD) is a process in which dying cells stimulate an immune response. It is a regulated form of cell death that can remodel the tumor microenvironment (TME) and activate the immune system, making immunotherapy more effective. This work was designed to identify prognostic gene features associated with ICD in cervical cancer (CC).

**Methods:**

Based on CC datasets and a set of ICD‐related genes obtained from public databases, we first filtered out ICD‐related genes unrelated to CC survival using univariate analysis. Subsequently, LASSO regression and multivariate Cox regression analysis were employed to develop prognostic feature genes based on ICD. For the construction and validation of the model, eight genes (CXCL1, IL1B, TNF, YKT6, PDIA3, ROCK1, CXCR3, and CLEC9A) were chosen. A nomogram was created to forecast the prognosis of CC individuals, and Kaplan–Meier curves were utilized to explore the survival disparities among different risk groups of CC individuals.

**Results:**

ssGSEA analysis was employed to investigate immune differences between two risk groups, revealing that the low‐risk group exhibited elevated levels of immune cell infiltration, enhanced activation of immune function, and a higher immunophenoscore compared with the other group, which highlighted the relevance of ICD to TME.

**Conclusion:**

We constructed a prognostic model based on genetic biomarkers of ICD for prognostic prediction of CC patients. Our model demonstrated excellent discriminative and calibration capabilities, providing a valuable tool for prognostic prediction and assessing the potential efficacy of immunotherapy in CC.

## 1 INTRODUCTION

Cervical cancer (CC) has the highest occurrence rate among the three main gynecological cancers and ranks fourth as a cause of cancer‐related deaths in women.^1^ According to the latest report from “Cancer Statistics, 2023,” there are still 13,960 new cases of CC and 4310 CC‐related deaths annually.^2^ Therefore, standardizing CC prevention, diagnosis, and treatment is crucial for improving women's health. The primary treatments for early‐stage CC mainly consist of surgery and radiotherapy, complemented by chemotherapy, targeted therapy, and immunotherapy.^3^ In recent years, the application of antiangiogenic targeted therapy and immune checkpoint inhibitors has offered new options for treating recurrent and metastatic CC.^4^ Hence, identifying novel biomarkers will aid in screening high‐risk individuals for recurrence and potentially benefit them in adjuvant therapy.

Immunogenic cell death (ICD) refers to a cellular process that triggers an immune response. It involves the transformation of tumor cells from being nonimmunogenic to immunogenic when exposed to external stimuli. This transformation enables the body to mount antitumor immune responses.^5^, ^6^ ICD, as a regulated form of cell death, can remodel the tumor microenvironment (TME) and holds the potential to activate adaptive immunity, thus facilitating the effectiveness of immunotherapy.^7^ Over the past few years, researchers have conducted numerous investigations on ICD. Among them, a substantial body of research has demonstrated the significant potential and advantages of ICD‐related biomarkers for forecasting the prognosis of cancer individuals. For instance, Li et al.^8^ confirmed the impact of ICD in breast cancer prognosis through their study. Similarly, Wang et al.^9^ analyzed public databases and discovered how ICD functions in the prognosis and immunotherapy of head and neck squamous cell carcinoma. However, the precise role of ICD in the prognosis and immune aspects of CC is still not fully understood. Therefore, conducting relevant research in this area holds significant clinical value in terms of predicting the prognosis and exploring the potential of immunotherapy for individuals with CC.

In this study, two distinct risk groups were identified based on ICD‐related genes in CC. Low‐risk (LR) group was linked with favorable clinical outcomes compared with the high‐risk (HR) group. The genes showing differential expression between different risk groups primarily participated in receptor‐ligand interactions, signal transduction, and activation of immune‐related signaling pathways, among other biological processes. Additionally, we built and validated an ICD‐related risk‐scoring prognostic model. The analysis demonstrated a remarkable association between this score and the TME of CC, indicating its independent prognostic value in predicting the outcomes of CC individuals. Overall, we established a novel CC prognostic model based on ICD, providing new solutions for prognostic prediction and treatment in CC.

## 2 MATERIALS AND METHODS

### 2.1 ICD genes in CC

**Figure 1:**
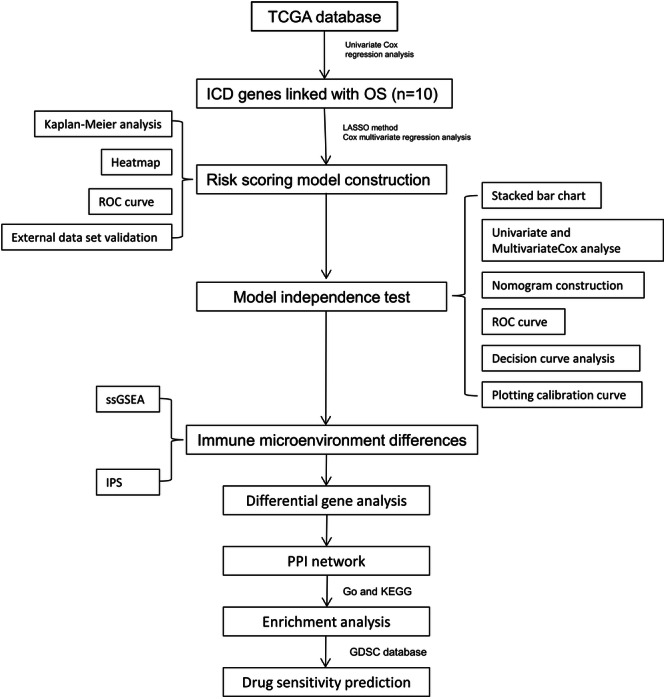
Flow chart.

Gene expression and clinical pathological data of CC were downloaded from The Cancer Genome Atlas (TCGA) database^10^ with a sample size of *n* = 309. For external validation, original gene expression data and clinical data were acquired from GEO database^11^ with a sample size of *n* = 300. ICD‐related genes were obtained from relevant literature.^8^ Univariate Cox regression analysis was fulfilled to initially identify ICD genes associated with overall survival (OS) in CC. The analysis process is shown in Figure 1.

### 2.2 Construction of a risk scoring model based on ICD

In TCGA data set, the range of survival‐associated ICD genes was narrowed down using the LASSO method, and the optimal parameter *λ* was adjusted through ten‐fold cross‐validation. The optimal cutoff value was computed utilizing the “survminer” package. The selected genes were subjected to Cox multivariate regression analysis to build the final prognostic model. The risk score for each individual was computed using the following formula:

Model=∑Coefficient(gene)*Expression value(gene).

The individuals' risk scores and survival outcomes were statistically analyzed, and the results were displayed using scatter plots. The data set was put into HR and LR groups as per the median risk score observed within the cohort. Kaplan–Meier curves were utilized to compare the potential survival rate differences between the two groups. The predictive performance was compared by assessing the differences in the area under the receiver operating characteristic (ROC) curve. Expression of prognostic feature genes in individuals was analyzed using a heatmap. Similarly, the aforementioned analyses were fulfilled in the validation set GSE44001 to rate the reliability, sensitivity, and specificity of the model.

### 2.3 Construction of nomogram based on the prognostic model and clinical features

Univariate and multivariate Cox regression analyses were fulfilled to assess the independent prognostic significance of the risk score. ROC curves were plotted, and the area under the curve (AUC) was calculated to evaluate the predictive ability of age, T stage, N stage, and established model.^12^ Subsequently, differences in clinical features (age, T stage, N stage, tumor stage) between the two groups were evaluated. Then, a nomogram was depicted by integrating the prognostic model and clinical factors determined by multivariate regression analysis. Calibration curves were depicted to rate the deviation between the nomogram and individuals' actual conditions. Predictive performance was assessed by calculating the ROC curve, and decision curve analysis (DCA) curve, and plotting the calibration curve.^13^

### 2.4 Analysis of immune microenvironment differences in different risk groups

To assay the immune infiltration level in each sample, we employed the “GSVA” package to perform ssGSEA. This analysis incorporated 28 gene sets obtained from previous studies^14^ and enabled a comparative assessment of immune infiltration across samples. The gene set consists of 782 genes was utilized to forecast the abundance of 28 tumor‐infiltrating immune cells in individual tissue samples. Additionally, expression of human leukocyte antigen (HLA)‐related genes and immune checkpoints in HR and LR groups was evaluated.

The immunophenoscore (IPS) has been reported as a reliable predictive factor for the response to immune checkpoint inhibitors (ICIs), like anti‐CTLA‐4 and anti‐PD‐1 treatments.^15^ It has demonstrated its ability to indicate potential responsiveness to ICIs in various types of tumors. Given the variations in the immune microenvironment status observed between HR and LR groups, we further examined the disparities in IPS between these two groups. IPS for each patient was obtained from The Cancer Immunome Atlas database.^15^

### 2.5 Protein–protein interaction (PPI) network and enrichment analysis

The network of differentially expressed genes (DEGs) between two groups was built utilizing the STRING database. Enrichment analyses (gene ontology [GO] and Kyoto Encyclopedia of genes and genomes [KEGG]) were fulfilled on DEGs using the “clusterProfiler” and “enrichplot” packages to elucidate potential functional pathways.^14^

### 2.6 Drug sensitivity prediction

Genomic data from the genomics of drug sensitivity in cancer (GDSC) database^16^ were utilized to forecast the drug sensitivity of CC cases included in the study. IC_50_ value was calculated utilizing the pRRophetic package to reflect the drug response.^17^

### 2.7 Statistical analysis

The primary endpoint analyzed here was OS, which was defined as the interval between the date of diagnosis and the date of death. Survival and ROC curves were depicted utilizing timeROC and survival packages, respectively. Nomogram was depicted using the “rms” package. All statistical tests conducted here were two‐sided, and a significance level of *p*‐value < .05 was used to determine statistical significance unless otherwise stated.

## 3 RESULTS

### 3.1 Construction and validation of the risk score model

**Figure 2:**
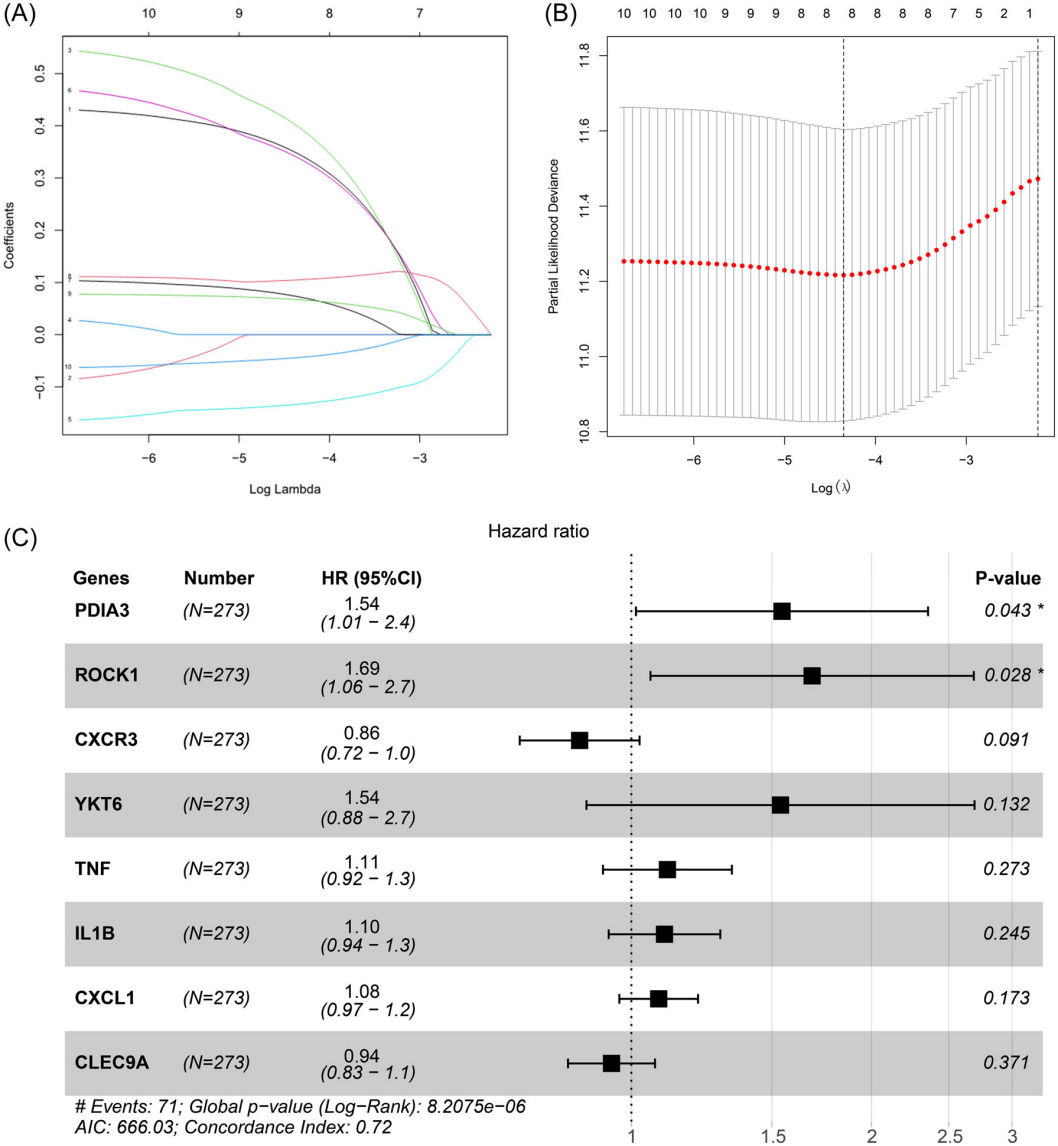
Screening of prognostic genes and construction of models. (A, B) Illustration of coefficient distribution (A) and partial likelihood deviance distribution (B) in LASSO analysis. (C) Multivariate regression displaying the model genes and their significance. *p < .05.

Fifty‐five ICD‐related genes were obtained from relevant literature. Through univariate regression analysis, 10 genes significantly linked with the survival of CC individuals were selected (threshold: *p* < .05) (Schedule S1). Furthermore, LASSO regression was employed to eliminate multicollinearity (Figure 2A,B). Ultimately, eight genes with nonzero coefficients were identified through multivariate regression and utilized to build the risk score model. Among them, CXCL1, IL1B, TNF, YKT6, CLEC9A, and ROCK1 were identified as adverse prognostic factors, while CXCR3 and PDIA3 as protective prognostic factors (Figure 2C). The formula for the model is as follows:

Riskscore=0.4348*PDIA3+0.5218*ROCK1−0.1492*CXCR3+0.4305*YKT6+0.1041*TNF+0.0956*IL1B+0.0789*CXCL1−0.0573*CLEC9A.

### 3.2 Validation of the prognostic model

In TCGA data set, CC individuals had varying risk scores, and higher risk scores were linked with poorer survival outcomes (Figure 3A). Heatmap of the feature genes showed significant upregulation of CXCL1, IL1B, TNF, YKT6, PDIA3, and ROCK1 in the HR group, while CXCR3 and CLEC9A exhibited significant downregulation in the HR group (Figure 3B). Survival curve analysis illustrated that individuals in the HR group had shorter OS in contrast to those in the LR group (Figure 3C). As ROC analysis revealed, the risk model had a good ability to stratify individuals into different risk groups (1‐year AUC: 0.83, 3‐year AUC: 0.71, and 5‐year AUC: 0.73) (Figure 3D). Similar findings were observed in the GEO data set (Figure 3E–H). In sum, these analyses demonstrated the successful development of an effective prognostic model based on ICD‐related genes in CC.

Figure 3Validation of prognostic models. (A) Scatter plot showing the survival outcomes and corresponding risk scores in The Cancer Genome Atlas (TCGA) samples. (B) Heatmap displaying the expression of prognostic feature genes in the TCGA samples. (C) Survival curve illustrating the survival rates of different risk groups in the TCGA data set, respectively. (D) receiver operating characteristic (ROC) curve presenting AUC values of 1‐, 3‐, and 5‐year in the TCGA individuals. (E) Scatter plot showing the survival outcomes and corresponding risk scores in GEO samples. (F) Heatmap displaying the expression levels of prognostic feature genes in GEO samples. (G) Survival curve illustrating the survival rates of different risk groups in GEO data set. (H) ROC curve presenting the AUC values of 1‐, 3‐, and 5‐year in the GEO individuals, respectively.

**Figure:**
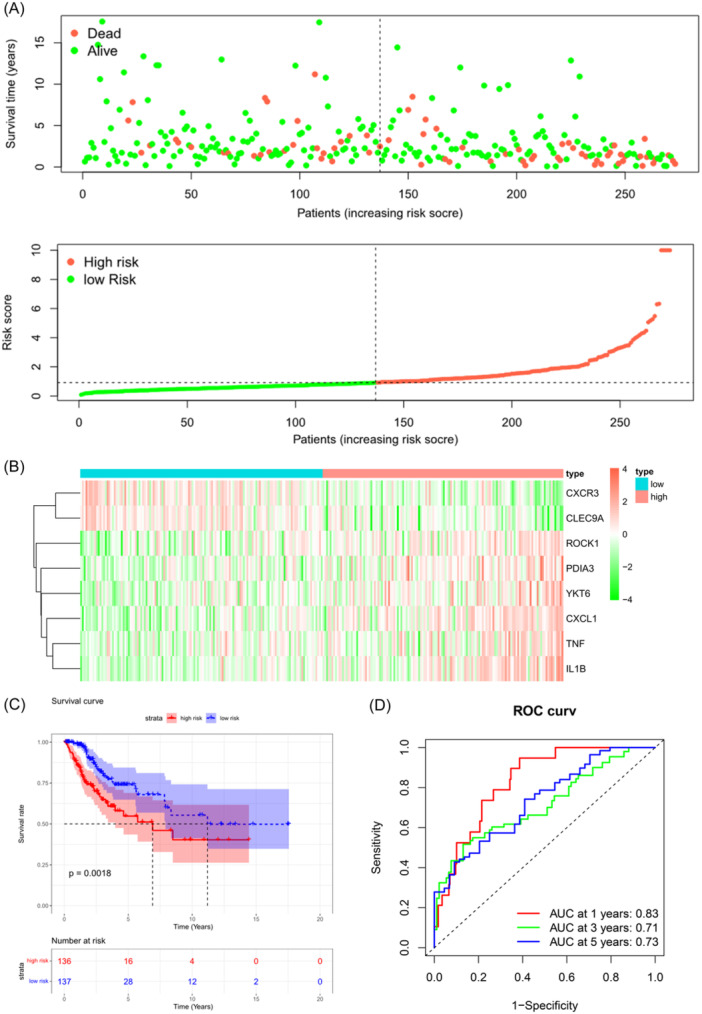

**Figure:**
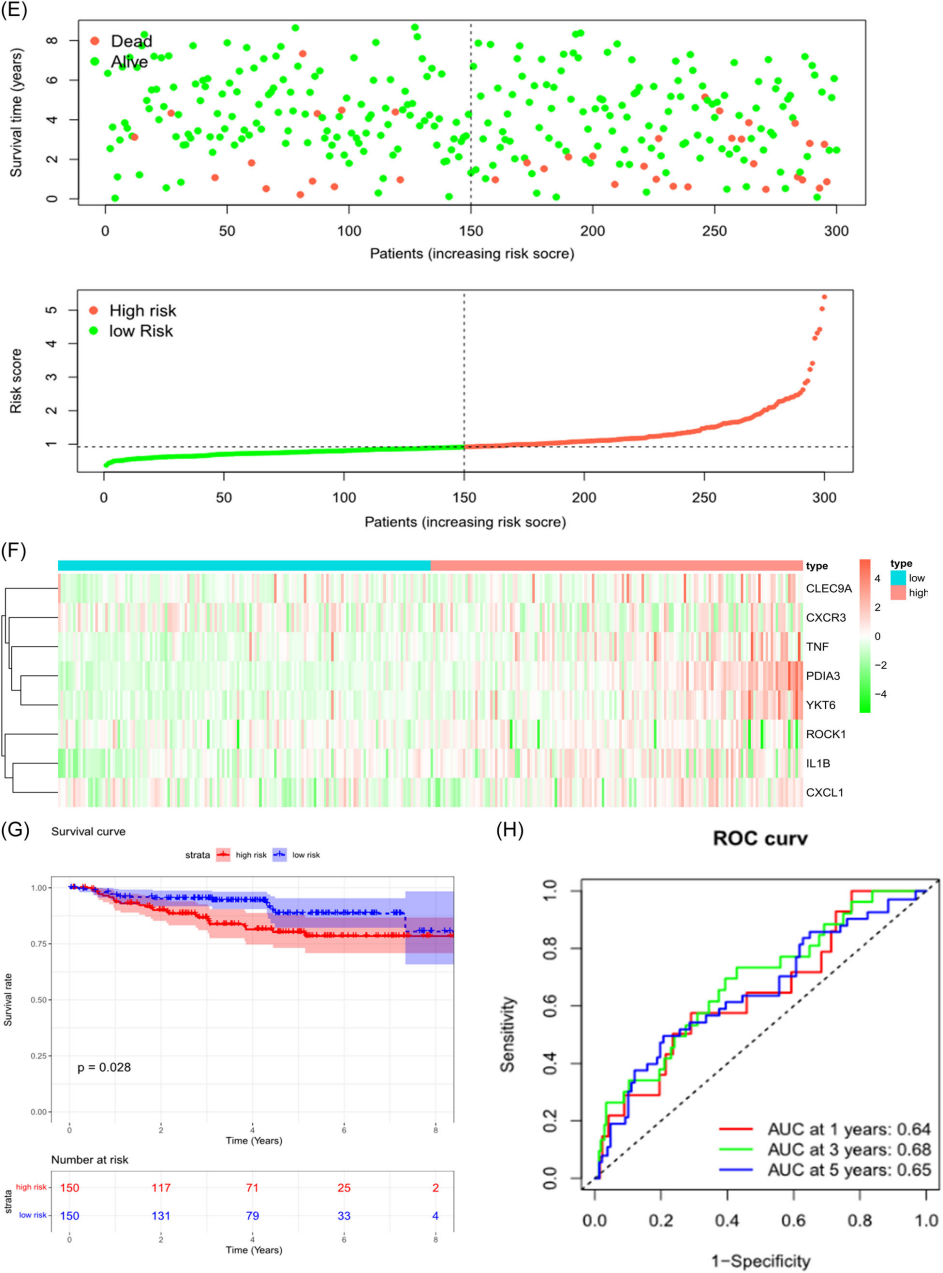

### 3.3 Independence test

**Figure 4:**
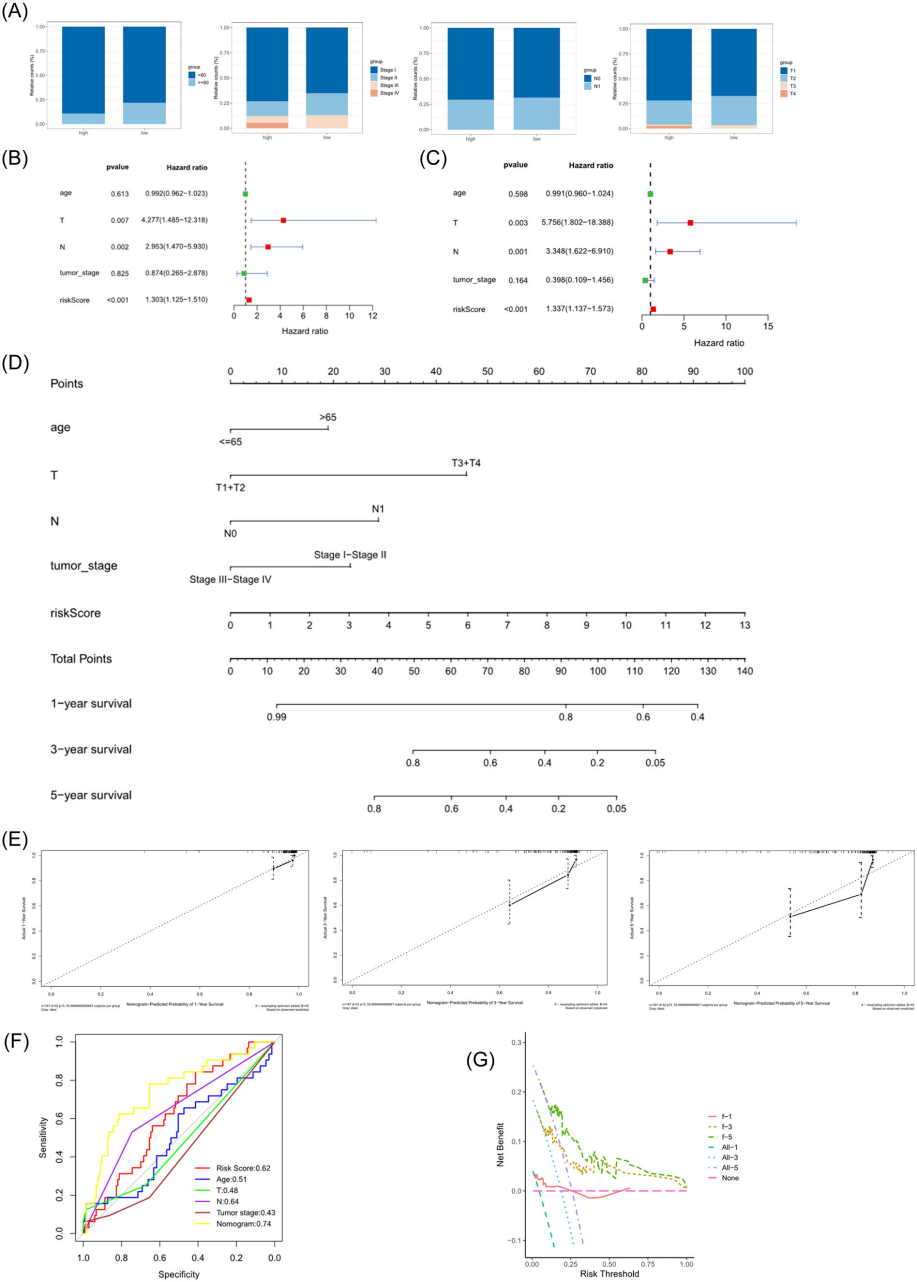
Verify the independence of the model. (A) Proportions of clinical features in the two risk groups. (B) Univariate analysis evaluating the independent prognostic performance of the model. (C) Multivariate analysis assessing independence. (D) Nomogram based on the model and clinical features. (E) Calibration curves of the nomogram for 1‐, 3‐, and 5‐year OS. (F) Receiver operating characteristic (ROC) curve of clinical features, riskscore, and nomogram. (G) Decision curve analysis curves of the nomogram.

By plotting stacked bar charts of different clinical features in two risk groups, it was observed that the HR group possessed a higher proportion of individuals under the age of 60. In terms of tumor stage, the HR group had a higher proportion of individuals in the T1 stage, T4 stage, stage I, and stage IV, while the difference between the N0 and N1 stages was not significant (Figure 4A). Furthermore, both univariate and multivariate Cox analyses ascertained that the risk score could independently forecast individuals' OS (Figure 4B,C). Subsequently, a nomogram was built by integrating the risk score and prognostic clinical features to forecast 1‐, 3‐, and 5‐year OS (Figure 4D). The calibration plot illustrated the good performance of the nomogram compared with the ideal model (Figure 4E). The generated ROC curve and DCA indicated the favorable predictive ability of the nomogram (Figure 4F–G). To sum up, these analyses suggested that the prognostic model based on ICD‐related genes can independently forecast patients' OS.

### 3.4 Evaluation of TME and immune cell infiltration levels in different risk groups

**Figure 5:**
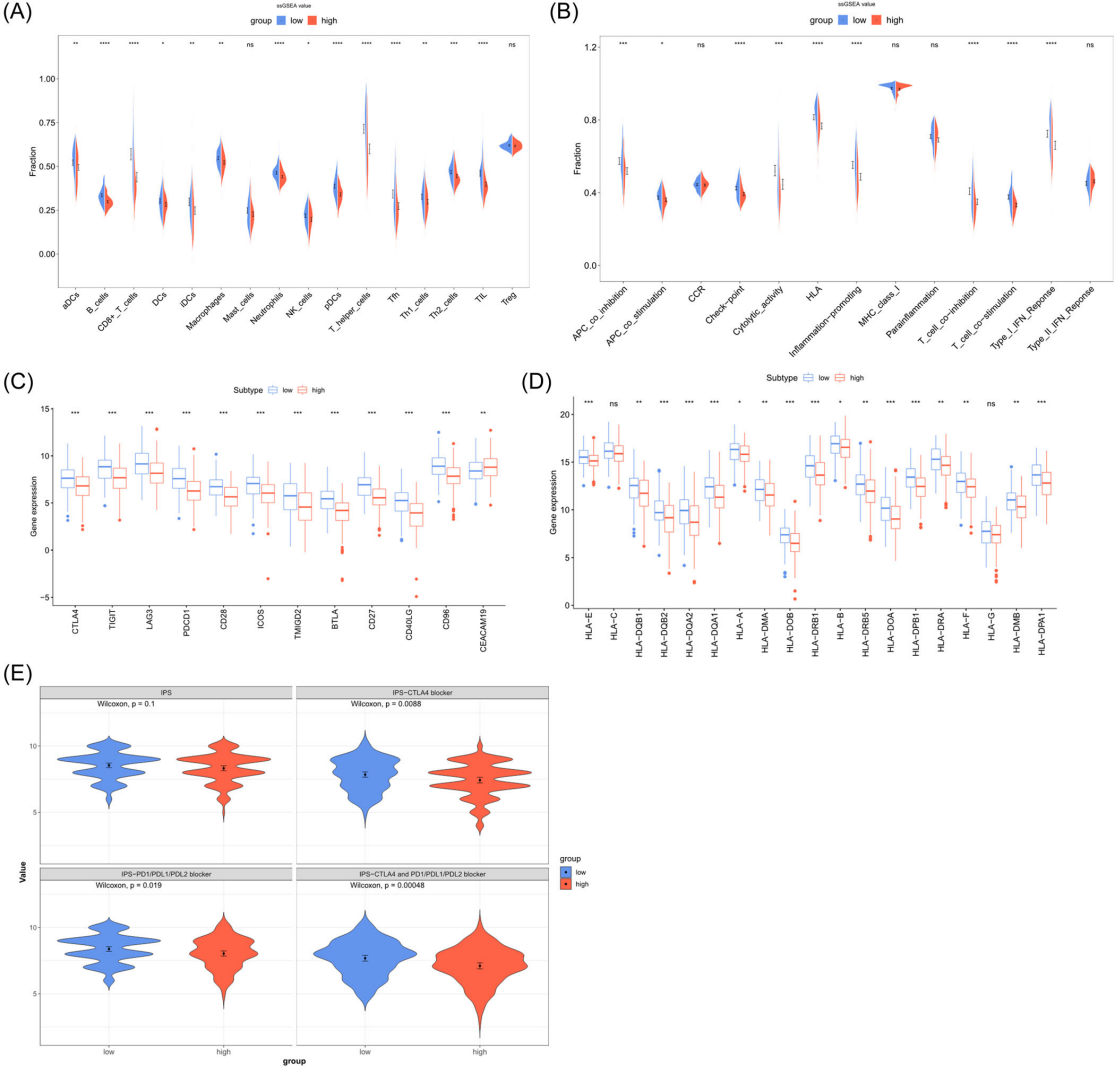
The immune microenvironment and immune cell infiltration levels are evaluated between the high and low‐risk groups. (A) Analysis of immune cell infiltration using ssGSEA. (B) Analysis of immune functional pathways using ssGSEA. (C) Expression of HLA genes in two risk groups. (D) Expression of immune checkpoints in two risk groups. (E) Immunophenoscore (IPS) between different risk groups. *p < .05; **p < .01; ***p < .001; ****p < .0001.

To dive into the disparities in immune status between the two groups, the enrichment scores of immune cells and immune‐related functional pathways were estimated utilizing ssGSEA. In TCGA cohort, except for Mast_cells and Treg, the levels of immune cell infiltration were tellingly decreased in the HR group (*p* < .05) (Figure 5A). Regarding immune‐related pathways, the HR group exhibited significant decreases (*p* < .05) in enrichment scores for APC costimulation/inhibition, T cell costimulation/inhibition, HLA, checkpoints, cytolytic activity, pro‐inflammatory response, and type I IFN response (Figure 5B). Additionally, differential gene expression analysis revealed higher expression of HLA genes and immune checkpoints in the LR group (Figure 5C,D). IPS analysis denoted that patients in the LR group possessed a favorable response to CTLA‐4, PD‐1, and CTLA‐4/PD‐1, suggesting their potential benefit from immunotherapy (Figure 5E).

### 3.5 Differential genes and functional annotation in different risk groups

**Figure 6:**
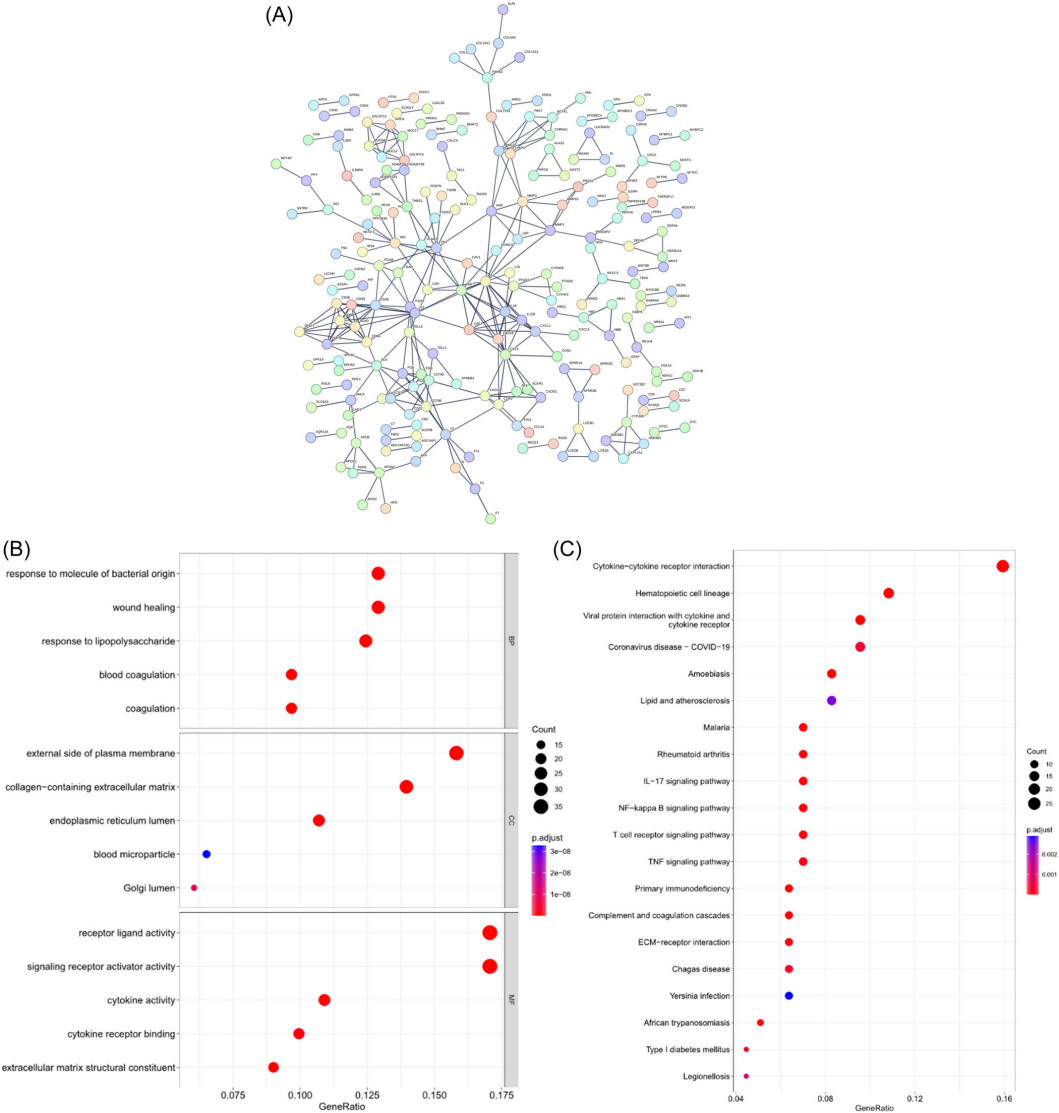
The differential genes between high and low‐risk groups are obtained and annotated functionally. (A) Protein–protein interaction (PPI) network illustrating the potential interactions among differentially expressed genes (DEGs) in the different risk groups. (B) Gene ontology (GO) enrichment analysis exploring the biological processes that may be regulated by the DEGs in the different risk groups. (C) Kyoto Encyclopedia of genes and genomes (KEGG) enrichment analysis investigating the signaling pathways that may be involved in DEGs in different risk groups.

By conducting differential analysis, we ascertained 894 genes that were differentially expressed between the different risk groups (Schedule S2). Utilizing the STRING database, a PPI network was constructed to visualize the potential interactions among these DEGs (Figure 6A). GO analysis revealed significant associations between these DEGs and biological processes like a response to molecules of bacterial origin, response to lipopolysaccharide, blood coagulation, receptor‐ligand activity, signaling receptor activator activity, cytokine activity, and extracellular matrix structural constituent (Figure 6B). As KEGG enrichment analysis indicated, the activation of pathways including cytokine‐cytokine receptor interaction, viral protein interaction with cytokine and cytokine receptors, IL‐17 signaling pathway, NF‐κB signaling pathway, T cell receptor (TCR) signaling pathway, tumor necrosis factor (TNF) signaling pathway, complement, and coagulation cascades, and ECM‐receptor interaction were significantly associated with these DEGs (Figure 6C). Combined, as per these findings, the activation of signal transduction mediated by receptor‐ligand interactions and immune‐related signaling pathways may be important factors contributing to the differential survival outcomes observed in the different risk groups.

### 3.6 Drug sensitivity analysis

**Figure 7:**
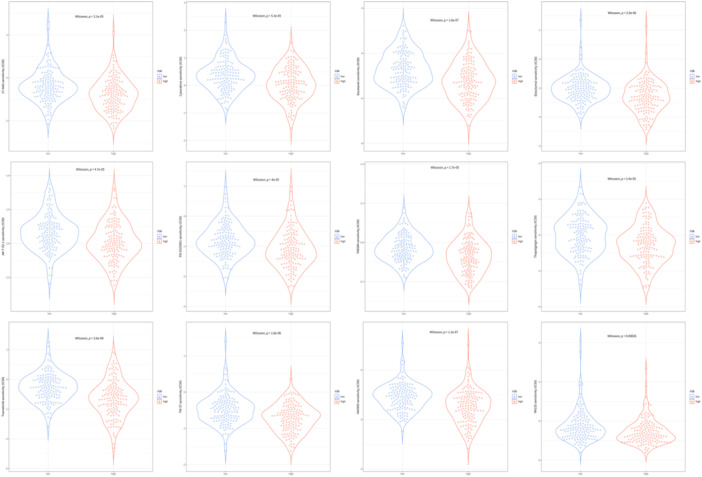
Predicting potentially sensitive drugs. Violin plots displaying the IC_50_ values of 12 drugs corresponding to different risk groups.

Violin plots were utilized to depict the IC_50_ values of 12 drugs corresponding to different risk groups, as forecasted based on the GDSC database. The outcomes exhibited significantly higher IC_50_ values for 17‐AAG, Cytarabine, Docetaxel, Elesclomol, Jw‐7‐52‐1, Pd‐0325901, Tae684, Thapsigargin, Trametinib, Tw 37, Xav939, and Ym155 in LR group compared with HR group (Figure 7). This indicated that individuals in the HR group were more likely to derive therapeutic benefits from these 12 drugs.

## 4 DISCUSSION

CC is a common malignancy that poses a threat to women's lives.^18^ Currently, insufficient screening remains a significant cause of the high incidence and mortality rates of CC. Therefore, timely prevention and diagnosis of CC are crucial for reducing its mortality rate. ICD is a regulated form of cell death involved in tumor progression.^7^ Nevertheless, the function of ICD‐related genes in the prognosis and immune microenvironment of CC is uncertain. Exploring the meaning of ICD‐related genes in CC prognosis holds important implications for improving survival rates.

Here, we ascertained the close linkage of ICD‐related genes with CC prognosis and the TME. We identified 8 feature genes that significantly impacted the OS of CC individuals and built a prognostic model based on the expression of these genes and their coefficients. Among them, CXCL1, IL‐1β, TNF, YKT6, PDIA3, and ROCK1 were found to be risk factors leading to poor prognosis, while CXCR3 and CLEC9A were prognostic protective factors. A study involving multiple tumors found that CXCL1 plays a tumor‐promoting role in reproductive cancers like breast cancer, CC, endometrial cancer, ovarian cancer, and prostate cancer.^19^ IL‐1β is an important member of the interleukin family, and Li et al.^20^ unmasked that upregulation of IL‐1β expression can activate colon cancer stem cells' self‐renewal and epithelial‐mesenchymal transition, thereby promoting colon tumor growth and invasion. TNF is a major pro‐inflammatory cytokine initially ascertained for its ability to trigger rapid hemorrhagic necrosis in experimental tumors.^21^ Recently, TNF has been recognized as a central cytokine driving inflammatory responses, both directly and indirectly, and biologic agents targeting TNF have become one of the most successful therapies for treating chronic inflammation and autoimmune diseases.^22^ Additionally, studies have shown that CXCR3 possesses high expression on activated T cells and plays a crucial role in spatial distribution, migratory behavior, and function of T cells.^23^, ^24^ Chow et al.^25^ found that mice lacking CXCR3 show poor response to PD‐1 blockade therapy, while mice with normal CXCR3 expression exhibit significantly reduced tumor growth after being treated with anti‐PD‐1 antibodies. Hence, our work's findings were in line with the notion that CXCR3 primarily exerts a protective effect. Furthermore, our study revealed that the risk‐scoring model composed of these eight characteristic genes exhibited good prognostic value in training and validation sets and may serve as a prognostic factor independently for CC individuals.

Besides, we observed a close linkage between ICD‐based risk scoring and the TME. Studies have demonstrated that both immunotherapy and chemotherapy trigger antitumor immune responses, including the expansion of CD8^+^ T cells.^26^, ^27^ Within the context of the cellular immune response, the proliferation and activation of T cells not only require the first signal provided by TCR recognition of antigen‐presenting cells (APCs) or tumor cells presenting MHC molecules but also rely on the second signal provided by costimulatory molecules.^28^ Here, we found that compared with the HR group, CC individuals in the LR group exhibited higher levels of CD8^+^ T cell and T helper cell infiltration, as well as increased activation status of APCs and T cells. Therefore, we hypothesized that the lower immune profile observed in the HR group may be linked with their poorer prognosis. Additionally, it is worth noting that in the assessment of immunotherapy response among different risk groups, individuals in the LR group demonstrated higher IPS compared with those in the HR group, indicating a favorable response to CTLA‐4, PD‐1, and CTLA‐4/PD‐1 blockade therapies, with potential clinical benefits in immune‐based treatments. These findings align with previous research results.^29^

Viewed in toto, this work highlighted the linkage between ICD‐based risk scoring and alterations in the TME. These observations may provide valuable insights for immune‐based interventions in CC individuals. We also screened ICD‐related prognostic features and constructed a prognostic model, which has a good ability to predict prognosis. However, limitations existed. First, our results need further validation in larger cohorts to define the cutoff values more precisely. Second, our analysis only discussed the influence of ICD‐related genes on CC prognosis and immune profile from a bioinformatics perspective, lacking a series of experimental validations. Therefore, this work aimed to forecast the potential linkage between ICD‐related genes and CC prognosis as well as immune therapy, offering a fresh perspective for prognostic prediction and clinical research in CC.

## AUTHOR CONTRIBUTIONS

Shuangmei Jing contributed to the study design, acquired the data, and wrote the manuscript. Jingdong Chen performed data analysis and revised the manuscript. All the authors gave the final approval of the version to be submitted.

## CONFLICT OF INTEREST STATEMENT

The authors declare no conflict of interest.

## Supporting information

Schedule S1: Using univariate COX regression analysis, 10 ICD‐related genes obtained from the literature are screened out to be significantly related to the survival of CC individuals.

Schedule S2: Differential analysis obtained 894 DEGs in different risk groups.

## Data Availability

The data used to support the findings of this study are available from the corresponding author upon request.
